# ICU outcomes in patients suffering granulomatosis with polyangitis

**DOI:** 10.1186/cc14615

**Published:** 2015-03-16

**Authors:** C Hernandez-Cardenas, GL Lugo-Goytia, MS Sierra Beltran, G Dominguez Cherit

**Affiliations:** 1 Instituo Nacional de Enfermedades Respiratorias, Mexico DF, Mexico; 2 Medica Sur, Mexico DF, Mexico; 3 Instituo Nacional de Ciencias Medicas y Nutrición Salvador Zubirán, Mexico DF, Mexico

## Introduction

This study aims to describe both the clinical course and prognostic factors of patients suffering granulomatosis with polyangitis (Wegener granulomatosis) (GP) who were admitted to the Salvador Zubirán National Medical Sciences and Nutrition ICU.

## Methods

Twenty-two patients suffering GP admitted to the ICU, between January 2002 and December 2012, were included. The Acute Physiology and Chronic Health Evaluation (APACHE) II prognostic score scale was used in order to assess the severity of illness on the first ICU day. The Sequential Organ Failure Assessment (SOFA) score was used to measure organ dysfunction, and the Birmingham vasculitis activity score for Wegener granulomatosis (BVAS/WG) was used to assess vasculitis activity. The outcome measurements taken into account were ICU mortality and ICU length of stay.

## Results

One patient was admitted twice during this period. The sample comprised 11 males and 11 females (50%, respectively). Featuring an average age of 52 years, 78% of them were admitted to the ICU because of respiratory failure, 50% were due to diffuse alveoli hemorrhage, 36% due to sepsis, 4% hypovolemic shock and finally 4% because of tuberculosis. According to the BVAS/WG, 20 patients corresponded to severe disease, one to limited diseases and one to persistent disease. The average ICU length of stay was 20.6 days and as inpatients 43 days. While comparing the SOFA score between alive and deceased patients there was a 0.5-point difference (*P *= 0.077), 63% of the alive patients were diagnosed while they were in the ICU. Plasmapheresis was found to be a protector factor (*P *< 0.05).

## Conclusion

The BVAS/WG score was significantly different between alive and deceased patients. Plasmapheresis was found to be a survival predictor. This study has shown that both SOFA and APACHE II scores have no prognostic value in these patients.

